# Allergen immunotherapy phase II trials: Challenges in dose finding 

**DOI:** 10.5414/ALX02033E

**Published:** 2019-12-30

**Authors:** J. Kleine-Tebbe, S. Kaul, R. Mösges

**Affiliations:** 1 Allergy and Asthma Center Westend, Berlin,; 2 Division of Allergology, Paul-Ehrlich-Institut, Langen, and; 3 Clinical Research International Ltd., Hamburg and Cologne, Germany

**Keywords:** allergen-specific immunotherapy, desensitization, dose finding studies, phase II studies, efficacy, allergen challenge

## Abstract

Phase II studies on allergen immunotherapy (AIT) should define the dose with the best balance between efficacy and safety (“optimal dose”). Their key role is based on dose selection for subsequent pivotal studies (phase III, field studies). Since products for AIT differ in composition and unit definitions, phase II trials are mandatory for new products and preparations being developed according to the German Therapy Allergen Ordinance (“Therapie-Allergeneverordnung”, TAV) due to current EMA guidelines since 2009. The latter permit various in-vivo models and endpoints for phase II studies, e.g., AIT-induced changes in skin test, nasal, conjunctival or bronchial provocation, or in exposure chamber or field trials. Selection and graduation of the doses, minimization of placebo effects, and sufficient numbers of patients are a challenge. Effort, required time, and costs are important variables for the initiators of phase II trials. Risks are characterized by e.g., a) too small doses without relevant differences compared to placebo, b) missing true dose-response relationships, c) strong placebo effect and consequently small “therapeutic window”, d) large heterogeneity and missing distinct differences (compared to placebo), e) too small effects in field studies due to low allergen exposure, f) missing dose-related increase (in case of too high doses). In the view of the Paul-Ehrlich-Institute, the unambiguous phase II trials with TAV products performed until today were not able to confirm the marketed doses for AIT. Regardless of the utilized model, more raw and single data should illustrate the individual outcome of AIT during phase II trials, facilitating an improved and more intuitive interpretation of the data (placebo effects? scattering?). In the medium term, evidence regarding AIT efficacy will considerably increase due to phase II trials as a prerequisite for subsequent phase III field studies. This affects all manufacturers offering AIT products in Germany and Europe.

**German version published in Allergologie, Vol. 41, No. 9/2018, p. 407-415**

## Introduction and definitions 

Clinical studies using allergen preparations have some special features that distinguish them from phase I and phase II studies with conventional drugs (e.g., small molecules): 

A. Phase I studies with pharmaceuticals are usually used to document safety in healthy subjects. Since the main side effects associated with the use of allergen preparations are allergic reactions to the allergen used, phase I studies in non-allergic individuals are not appropriate to investigate safety. In addition, they are not possible due to ethical reasons (risk of sensitization). Thus, phase I studies with various application forms of preparations for allergen immunotherapy (AIT) [1] are already carried out in allergic subjects. 

B. The term “phase II study” usually refers to clinical investigations in which an active substance (pharmacon) is tested in patients who suffer from the investigated disease. This does not apply to AIT products, as safety testing in allergic patients with regard to the maximum acceptable dose already was carried out in the previous phase I studies. 

**Figure 1. Figure1:**
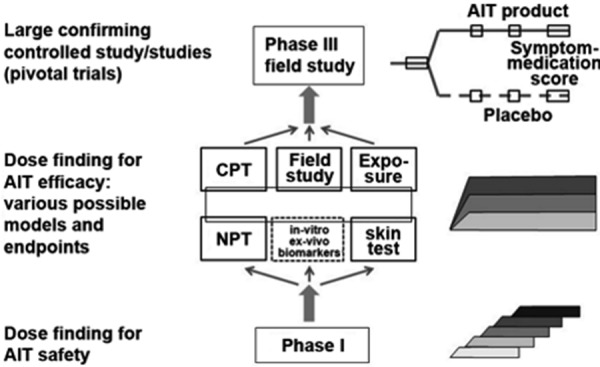
Clinical development program for allergen immunotherapy. Center: Possible models and methods for phase II dose finding studies on efficacy. Additional laboratory parameters or biomarkers (dotted box) are optional but not sufficient as study endpoints. AIT = allergen immunotherapy; CPT = conjunctival provocation test; NPT = nasal provocation test; Exposure = provocation in exposure chamber.

Thus, phase II studies fulfill another important task in AIT investigation: to find the dose with the best efficacy-safety ratio (“optimal” dose).[Fig Figure1]

## AIT dose finding in the past 

In the course of the more than 100-year history of AIT, already the pioneers have probably thought about the right dose: On the one hand, the effectiveness of AIT would have to be guaranteed, and, on the other hand, the preparation administered to allergic patients would have to be safe and with justifiable side effects. 

In the past, the dose-dependent effects of AIT were only rarely assessed in prospective studies (overview in [2]). Instead, the effective allergen quantity was calculated retrospectively based on the administered dose, when clinical studies had been successful [3]. It is only since the turn of the millennium and the change towards the intention to develop AIT products as systematically as other pharmaceuticals, that AIT products are tested prospectively and dose-dependently in phase I and phase II studies before large, multi-center, controlled phase III pivotal trials are to confirm their efficacy and safety. In particular, tablet products containing grass, ragweed and mite allergens for sublingual AIT have been tested in this way over the past 15 years, setting new standards for the clinical development of AIT products. 

## Arguments for AIT dose finding studies 

AIT preparations differ in their qualitative as well as quantitative composition. This refers to both the spectrum and the amount of detectable allergens. In Europe, there are currently no general standards (biological reference preparations (BRP)) based on which the manufacturers would have to label their products, as they have to in the USA, for example. Instead, all manufacturers define their own standards for each allergen source (in-house reference preparations (IRP)) using methods for biological standardization (titrated prick or intradermal tests) and various laboratory tests (physicochemical, biochemical, and immunological procedures) [4]. 

Based on the internal characterization of the allergen preparations, in-house units are defined by the manufacturer in order to be able to standardize future products referring to the IRP. As a consequence, the preparations are not comparable between manufacturers – neither in allergen composition (qualitatively and quantitatively) nor regarding units. 

For this reason, studies on the safe and effective dose of an AIT preparation cannot be transferred to other products. Thus, the safe and effective AIT dose has to be evaluated separately for each product. 

## Regulatory framework for dose finding studies 

According to European law, allergen preparations for diagnosis and treatment are medicinal products. This leads to regulatory requirements specified in two guidelines issued by the European Medicines Agency (EMA). 

A. The EMA guideline on product quality [5] contains valuable information on the characterization and standardization of natural extracts. In addition, the “principle of homologous groups” was introduced and defines related allergen sources (e.g., birch, hazel, and alder pollen or Poaceae pollen other than maize) based on important structurally similar major allergens [5]. 

B. The EMA guideline on clinical development [6] explains how to clinically test the safety and efficacy of AIT preparations in a stepwise manner. The regulatory requirements for the necessary phase II dose finding studies (DFS) on efficacy and safety are large. Since knowledge on the optimal dose finding for AIT preparations is lacking, the manufacturers can choose their preferred methods to test a potentially effective and safe dose. However, the guideline clearly states that using exclusively laboratory values (“biomarkers”) does not suffice for dose finding. Instead, clinical parameters like skin tests, nasal, conjunctival, or bronchial provocation test, exposure chamber, or field studies should be used. 

Most often, clinical data are supplemented by extensive laboratory testing to gather additional information on immunologic changes and possible biomarkers in DFS. 

## Requirements for dose finding studies 

The above-mentioned EMA guideline on clinical development [6] allows for considerable variance with regard to design and conduct of DFS. However, over the last 10 years, some requirements and framework conditions have emerged which have been discussed by the manufacturers and the competent authorities (Paul-Ehrlich-Institute or other international agencies responsible for marketing authorization, e.g., EMA). ([Table Table1])

The doses tested for safety, efficacy, and tolerability are usually compared to placebo. A statistically significant difference is not necessarily required, but there should be a clear numerical difference at least compared to placebo and ideally also between the different dosages tested. Thus, trends between the different allergen doses, which can theoretically also be made visible with small case numbers, are sufficient. However, the determined allergen dose with an optimal safety-efficacy relationship might not prove itself in the subsequent phase III field study. For clinical development it is therefore advisable to conduct phase II studies with a sufficiently large number. This is the only way to compensate issues caused by individual variability and inevitable placebo effects (see below). 

**Figure 2.**
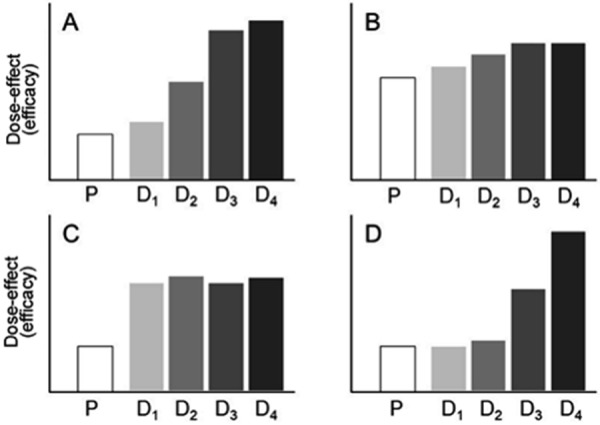
Examples of result patterns in dose finding studies (phase II) on efficacy. Preparations for allergen immunotherapy: A: Clear dose-dependent effects with plateau formation when the highest dose is used (D4); B: Small therapeutic window and minor differences due to high intervention/placebo effect (P); C: No dose-response relationship, probably due to too high doses; D: Dose-dependent effects without plateau formation so that most effective dose cannot be determined. P = placebo control; D1 – D4 = increasing doses of the AIT preparation.

Furthermore, the number of tested allergen doses must be high enough and should also include doses higher than those currently on the market; the latter is particularly important for AIT products developed according to the German Therapy Allergen Ordinance (“Therapie-Allergeneverordnung” (TAV)). In theory, this is only possible using four different doses. In addition, plateau formation regarding efficacy, which suggests a lack of increase in efficacy using higher doses, is ideal when various doses are compared (Figure 2). 

## Current state of dose finding studies 

Obtained data are, after completion of DFS and after having been evaluated,

- submitted to the authorities responsible for marketing authorization,
- concisely presented to European (https://www.clinicaltrialsregister.eu/) or US (https://clinicaltrials.gov/) databases, and
- frequently published (e.g., as abstracts on conferences, as original manuscripts in scientific journals).

As the publications lag behind the evaluation and only selected information is published, the approving competent authorities generally have the most comprehensive information on the so-far evaluated DFS. The data presented in Table 2 are therefore incomplete and only able to reflect published data. 

According to the Paul-Ehrlich-Institute none of the marketed dosages has been confirmed as the “optimal” dose in the clearly evaluable DFS of TAV products. 

Different patterns can be seen in DFS (Figure 2): for example,

- strong placebo effects,
- insufficient effects compared to placebo,
- lack of dose-effect relationships, or
- incomplete dose-effect curves (without efficacy plateau or limiting safety parameters).

These observations indirectly underline the necessity of systematic DFS with AIT preparations. If a higher dose proves to be effective and at the same time remains safe, this higher dose – instead of the currently marketed dose – should be tested in a subsequent phase III field study. 

Only little is known about the pharmacokinetics of allergen application [1, 7]. In particular, it is unclear which time interval between the allergen applications in SLIT or SCIT is ideal to efficiently induce immunological processes. The spectrum of used protocols (daily, (several times) weekly, monthly), method of updosing, and/or type of the adequate adjuvant leaves room for further treatment optimization studies in dose finding. 

**Figure 3.**
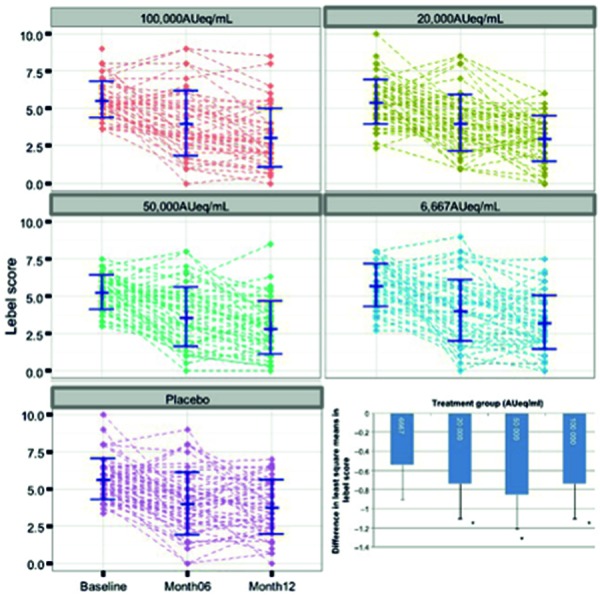
Individual results of a dose finding study using a house dust mite allergoid for SCIT (Pfaar O et al. Allergy 2016; 71(7): 967-76; cf. corresponding online data) [8]. Individual data (course in the “Lebel symptom score” after nasal provocation, y-axes) of all subjects, grouped according to SCIT dose or placebo (displayed above the graphs) before start (baseline), after 6 and 12 months (cf. x-axis on the bottom left). Bottom right: Presentation (same data set) of the group mean values compared to placebo (= 0) after 12 months of SCIT

Unfortunately, only averaged values (i.e., means, medians) of AIT products are often presented in scientific publications on DFS. Even if they contain additional standard deviations or confidence intervals, they make intuitive assessment of individual data scattering difficult (example in Figure 3 [8]). Future publications should therefore contain more individual data from both placebo-treated and actively treated subjects. This would lead to a higher transparency of DFS results, i.e., the (inevitable) placebo effects, the absolute and relative differences between dosages, and the individual response of subjects would become clearer. 

## Value of and outlook on dose finding studies with allergen immunotherapy products 

The years 2010 to 2020 might later be called the phase of “systematic dose finding for allergen immunotherapy”. Since current regulations force all manufacturers that develop products for the European market to test the most important allergen sources in a stepwise approach, valuable data, e.g., on dose finding, are generated that are not only relating to safety but also, and particularly, to efficacy. This closes an important gap that often remained in the past in the recommended dosage of AIT products. 

Thus, consistent and successful DFS are therefore able to

- establish the dose-dependent efficacy of an AIT product,
- allow approximation towards an optimal dose with an acceptable balance between safety and efficacy, and
- meet the prerequisite for a large-scale phase III field study by allowing to choose a justified dose.

Since pivotal trials involving symptom and drug use assessment entail effort, costs, and risks for the manufacturers, the preceding DFS plays an important role, as the most successful and later-on possibly approved dose, which is to be tested in at least one field study, will be selected here. 

## Conclusion

- DFS are the basis for subsequent pivotal trials and the range of AIT products that will be available in Germany in the future.
- It is to be expected that DFS on AIT preparations will continue to use different and difficult to compare models in the near future.
- Current efforts concern the standardization of and consensus reports on (phase II/III) study endpoints [9], provocation testing [10], pollen exposure times [11], and the use of allergen challenge chambers [12].
- Data obtained in parallel in-vitro and ex-vivo investigation can accelerate the development of suitable biomarkers to monitor the success of an AIT.
- The latter can currently not replace DFS in-vivo models [13].
- In the medium and long term, DFS will significantly increase the evidence on dose-dependent efficacy of AIT.

## Acknowledgment 

We thank Doris Ruhland and Vera Wisliceny for writing down the manuscript and Sophie Wirth for her careful corrections. 

## Conflict of interest 

Author^1^ and lecturer fees^2^, research support^3^, and advisory fees^4^: 

Allergen Online^4^, Allergopharma^1,2,3^, Allergy Therapeutics^4^, ALK-Abelló^2,3,4^, AstraZeneca^2^, Bencard^2,4^, Dr. Pfleger^2^, Dustri-Verlag^1^, Glaxo^3^, HAL Allergy^2,3^, InfectoPharm^2^, Leti^2,3,4^, Lofarma^2,4^, Novartis^2,3,4^, Merck (US)^ 4^, Parexel International^3,4^, Roxall^2^, Sanofi^2^, Stallergenes-Greer^2,3^, Springer International/Medizin^1^, ThermoFisher^2^, Georg Thieme Verlag^1^, WHO/IUIS Allergen Nomenclature Subcommitee^4^. 

**Table 1. Table1:** Requirements, challenges, and consequences/risks of dose finding on the efficacy of preparations for allergen immunotherapy.

Requirements^1^ for dose finding studies	Challenges^2^ imposed by dose finding studies	Consequences/risks^3^ of dose finding studies
- Use of an in-vivo model and suitable endpoint (not only in-vitro and/or ex-vivo data) - Testing of an adequate dose range - Testing of ≥ 3 doses, e.g., below or above the marketed dose - Significant difference compared to placebo - Differences (clear trends) between the doses; but statistically significant differences not required	- Use of a suitable model, e.g.: titrated skin test (e.g., intradermal test with late-reading) • conjunctival or nasal provocation test • allergen challenge in exposure chamber field study (symptom and medication scores) - Selection and scaling of doses (doubling? triplication? semi-logarithmic?) - Minimization of placebo (intervention) effect - Sufficient number of cases (power calculation) - Study costs and efforts	- Too low doses (no relevant differences) - No real dose-response relationship, no plateau - Too small therapeutic window when pronounced placebo effect is present - When scattering is too big, possibly no clear difference (compared to placebo) - Lack of treatment effects in field studies due to lack of allergen exposure - Missing dose increase (when doses are too high) - Repeat dose finding if results are ambiguous or negative

^1^Requirements are defined by the EMA guideline on the clinical development of AIT products [6], and compliance is monitored by the competent regulatory authorities (e.g., Paul-Ehrlich-Institute, Langen) based on the submitted study protocols. ^2^Challenges refer to study planning, design, and decisions before the study starts. ^3^Consequences/risks refer to possible impacts after conduct and evaluation of the phase II study.

**Table 2.** Published dose finding studies (allergen immunotherapy) carried out in line with the German Therapy Allergen Ordinance (TAV) or independently thereof.

**Application/Ref.**	**Allergen source**	**Allergen preparation**	**Model**	**Result(s)**	**Comment**
TAV allergens					
SLIT [14]	Birch pollen	Drops (non-modified)	NPT	With highest doses, statistically significant improvement compared to placebo after 5 months	Marked placebo effect (approx. 30%); no plateau formation, largest difference to placebo with highest dose
SCIT [15]	Birch pollen	Allergoid with adjuvant	CPT	2 dose finding studies (comparison of cumulative dose) with symptom reduction	Absolute and relative differences significantly better compared to placebo with plateau formation with highest dose
SCIT [15]	House dust mite	Allergoid	NPT	With higher doses, statistically significant improvement compared to placebo after 12 months	Moderate differences due to marked placebo effect (approx. 30%) and considerable data scattering (Figure 3)
SLIT [16]	Grass pollen	Tablet (Allergoid)	CPT	No placebo group but 4 graduated actively treated groups. Significant superiority of marketed dosage according to patient assessment (secondary parameter)	No consistent dose-response relationship in primary endpoint. Interpretation of data difficult because no placebo was used.
SLIT [17]	House dust mite	Tablet (Allergoid)	CPT	Only one dose was statistically significantly superior to placebo	Small therapeutic window and only minor differences due to high placebo effect (approx. 50%)
Non-TAV allergens					
SLIT [18]	Bet v 1	Tablet (recombinant, non-modified)	Field study	All 3 doses statistically significantly superior to placebo	No real dose-response relationship
SCIT [15]	Bet v 1 FV (folding variant)	Modified	Exposure chamber	All 4 doses statistically significantly superior to placebo	No real dose-response relationship
SCIT [20]	Bet v 1 peptides	Peptide immunotherapy	Field study	Only 2 concentrations tested against placebo; only smaller dose statistically significantly superior to placebo	Higher dose lower effect but more side effects; too few doses for real dose-response relationship
SCIT [21]	Lolium peptides	Peptide immunotherapy	CPT	Medium dose in responder analysis statistically significantly superior to placebo	Dose-response relationship with plateau reached in responder analysis
SCIT [22]	Timothy grass	Allergoid	IDT (LPR)	All doses statistically significantly superior to placebo	Significant improvement only in primary endpoint (IDT) without clear dose-response relationship; in exposure chamber, symptoms not significantly better compared to placebo

The listed phase II studies to define the optimal dose for allergen immunotherapy illustrate the used products, models, results, and interpretations but may not be complete. Bet v 1 = birch pollen major allergen; CPT = conjunctival provocation test; IDT = intradermal test; LPR = late-phase reaction, delayed phase of immediate-type reaction; NPT = nasal provocation test; SLIT = sublingual immunotherapy; SCIT = subcutaneous immunotherapy.
